# Longitudinal assessment of growth velocity in relation to pubertal timing, IGF-1, fasting insulin and fat mass in healthy boys

**DOI:** 10.1530/EC-25-0667

**Published:** 2026-02-23

**Authors:** Kaspar Sørensen, Casper P Hagen, Lise Aksglæde, Rikke Beck Jensen, Anders Juul

**Affiliations:** ^1^ Department of Growth and Reproduction, Copenhagen University Hospital – Rigshospitalet, Copenhagen, Denmark; ^2^ International Center for Research and Research Training in Endocrine Disruption of Male Reproduction and Child Health (EDMaRC), Rigshospitalet, University of Copenhagen, Copenhagen, Denmark; ^3^ Department of Paediatrics, Herlev University Hospital, Herlev, Denmark; ^4^ Department of Clinical Medicine, University of Copenhagen, Copenhagen, Denmark

**Keywords:** boys, puberty, growth, insulin, IGF-1, fat mass

## Abstract

**Background:**

Age at puberty determines the timing of pubertal growth spurt. Whether the intensity of the peri-pubertal growth velocity is also affected by pubertal timing in boys remains to be elucidated.

**Objective:**

To study changes in growth velocity in relation to pubertal timing, insulin-like growth factor 1 (IGF-1) and fasting insulin in healthy boys.

**Design, setting and participants:**

This is a longitudinal study with biannual assessment of testicular volume (TV), growth velocity, IGF-1 and fasting insulin levels. The peak height velocity (PHV) was calculated. 105 boys (947 examinations) were included. Pubertal onset was available in 62 boys – the rest remained pre-pubertal throughout the study period or were in puberty at baseline.

**Results:**

Age at pubertal onset (TV > 3 mL) was negatively correlated with PHV (*ρ* = −0.48; *P* < 0.001). The early (age of onset <33.3 percentile) tertile of maturing boys had a significantly higher growth velocity (mean Δ 0.48 (0.14–0.82) cm/year; *P* < 0.006) than late maturing boys (>66.7 percentile) over the 6-year peri-pubertal period. However, the late maturing boys remained significantly taller throughout the study (*P* < 0.05). IGF-1 levels were similar between the three groups of boys. In all boys, the increase in growth velocity was associated with a larger increase in IGF-1 (*P* < 0.001) during the first 2 years of puberty.

**Conclusion**

Early maturation was associated with an increased growth velocity and PHV in healthy boys. However, it was not sufficient to compensate for the shorter total growth period. IGF-1 was positively associated with growth velocity.

**ClinicalTrials.gov ID**

NCT01411527.

## Introduction

Age at sexual maturation has declined over the recent decades (1, 2, 3, 4) concomitantly with a tendency to becoming taller in the general population (5, 6, 7). The fact that pubertal onset seems to be more affected than later pubertal milestones implies that the pubertal period has become relatively longer, while the total linear growth period has become relatively shorter.

In healthy children, early maturation is associated with augmented growth and peak height velocity (PHV) during puberty (8, 9). In girls, age at pubertal onset is inversely associated with the time span from pubertal onset to PHV (10, 11). These factors seem to fully compensate for the earlier closure of the epiphyseal growth plate, leaving early-maturing girls at a similar final height as late-maturing girls (11), but data in boys are lacking.

The growth hormone (GH)/insulin-like growth factor 1 (IGF-1) axis is of paramount importance to linear growth in children and adolescents (12). In addition, insulin, which shares many similar growth-promoting properties with IGF-1 (13), may also be involved. Although GH directly stimulates the production of IGF-1 and indirectly increases insulin levels by inducing insulin resistance, all three hormones engage in a delicate interplay to maintain homeostasis (14).

Pre-pubertal IGF-1 levels are associated with faster growth rates before puberty (15) as well as earlier pubertal onset (16) in both boys and girls as well as earlier age at menarche in girls (17).

Higher insulin levels are related to a higher growth velocity in a mixed cross-sectional cohort of pre-pubertal and pubertal boys and girls (18). In addition, early pubertal onset is associated with higher insulin levels during puberty (11, 19), and in girls, the increase in insulin levels predicts augmented growth velocity during puberty (11). However, to what extent insulin levels are related to pubertal timing and growth velocity in boys is not well known.

In addition to IGF-1 and insulin, adiposity may also have an effect on growth velocity. Previous studies have found that adiposity increases linear growth during childhood at the expense of a more tempered pubertal growth spurt; thus, adiposity through childhood and adolescence does not seem to increase final height (20, 21).

The aim of the present study was to evaluate PHV and changes in growth velocity in relation to pubertal timing, skin fold thickness (SFT), IGF-1 and fasting insulin concentrations in healthy peri-pubertal boys followed longitudinal for 6 years.

## Materials and methods

### Study subjects

In this longitudinal study, as part of the COPENHAGEN Puberty Study (ClinicalTrials.gov ID: NCT01411527), we examined 105 healthy Danish boys every 6 months over the course of 8 years (2006–2014). A total of 947 examinations were carried out, and the median age at first examination was 9.0 years (range, 5.8–12.6 years). The median (range) number of examinations was 10 (2–14). All participants were recruited from two schools in the Copenhagen area. The schools were in the upper 20% with respect to parental income and socioeconomic status in a national investigation. All the examined boys were healthy. Other aspects of the COPENHAGEN Puberty Study have previously been published (2, 3, 22, 23).

### Clinical examination

Height was measured to the nearest 0.1 cm using a stadiometer (Holtain Ltd, UK). Weight was measured to the nearest 0.1 kg using a digital electronic scale (SECA). The children were weighed without shoes, wearing light clothing. Body mass index (BMI) was calculated as weight divided by height squared (kg/m^2^). BMI percentile-for-age was calculated according to the Center of Disease Control and Prevention 2000 (CDC). Overweight was defined between the 85th and 95th percentile-for-age, and obesity was defined above the 95th percentile-for-age. The thickness of four skinfolds (biceps, triceps, subscapular and suprailiac crest) was measured on the left side of the body using a skinfold caliper calibrated to 0.2 mm (Harpenden, British Indicators Ltd, UK). The sum of all skin folds (biceps, triceps, supra-iliac crest and subscapular) was calculated. Growth velocity was calculated at every half-year visit as the difference between the current height and the height half-year previous times 2.

All pubertal evaluations were performed by the same three physicians. Testicular volume (TV) was evaluated with an orchidometer on both testes. Genital stages (G1–G5) and pubic hair (PH1–PH5) were assessed by clinical examination according to Marshall and Tanner in all boys (24). Although the age midway between the examination before and the examination after reaching a TV of greater than 3 mL would be a more accurate estimate of age at pubertal onset, all the other variables (anthropometrics and hormone levels) were measured at a visit, and not in between visit. Thus, we decided to define pubertal onset as the first examination with a TV of more than 3 mL. The testes with the highest volume counted. A TV of more than 3 mL could be determined in 62 boys with a total of 766 biannual examinations spanning 9.5–15.5 years. The boys were divided into tertiles of early, middle and late age at pubertal onset based on the 33.3 and 66.7-percentiles (early *n* = 21, middle *n* = 21 and late *n* = 20).

### Blood sampling procedure

Fasting blood samples were drawn from an antecubital vein between 08:30 and 09:30 h. Blood samples were clotted and centrifuged, and serum was stored at −20°C until hormone analyses were performed. Of the 777 examinations were 699 blood samples available for IGF-1 measurements and 524 blood sample available for fasting insulin measurements. Glucose was measured in 262 blood samples.

### Serum hormone analyses

Serum concentrations of insulin and glucose were determined by an electrochemiluminescence immunoassay (Elecsys insulin reagents kit; Roche Diagnostics, Germany) on an automated Roche Modular Analytics Module E170 (Roche Diagnostics). The detection limit for insulin was 2 pmol/L, and the intra- and inter-assay CV values were 4.2 and 8.2%, respectively. Glucose was determined on automated Roche Modular Analytics Module P (Roche Diagnostics) by enzymatic absorption photometry (GLU, Roche, Germany) with intra- and inter-assay CV values < 2%, respectively. Serum IGF-1 and IGF-binding protein (IGFBP)-3 were determined by immunoassay (IMMULITE 2000, Siemens Healthcare Diagnostics, Germany) with detection limits of 20 and 100 ng/mL, respectively. IGF-1 intra- and inter-assay CV values were 2.1 and 10.1%, respectively; for IGFBP-3, CV values were 4.3 and 9.2%, respectively.

### Statistical analyses

Data are presented as mean and 95% confidence intervals (CIs). Simple cross-sectional group comparisons were done with one-way ANOVA. Simple correlations were evaluated with Spearman rho correlations (*ρ*).

Differences in height, growth velocity, BMI, sum of skinfolds, IGF-1, IGFBP-3 and fasting insulin levels between the three tertiles of early-, middle- and late-maturing boys were evaluated by linear mixed models to account for the repeated measurements on each individual. Models were done with autoregressive AR(1):heterogeneous as repeated covariance type. Models were done with either chronological age (half-year intervals) from age 9.5 to 15.5 years or adjusted age from the onset of TV > 3 mL (half-year intervals) from 3.0 years before to 3.0 years after the onset of puberty. The dependent variables were log-transformed to obtain normal Gaussian distribution of the residuals. Tertiles of early, middle and late onset of puberty and age/corrected age were included as fixed factors. Results are shown as mean differences and 95% CIs between the maturation groups.

In order to evaluate the longitudinal changes in height, SFT, IGF-1 and fasting insulin from each individual before and after pubertal onset, we calculated the slopes by linear regression. In order to not violate the rules of linearity, we choose −2 to 0 years before and 0–2 years after pubertal onset for height, SFT, IGF-1 and insulin. Boys with at least 4 of the 5 measurements in each analysis were included. We expanded SFT periods to −3 to 0 and 0–3 years from pubertal onset in that these periods did not violate the rules of linearity. Boys with a minimum 5 of 7 measurements were included. Associations were done by univariate ANOVA.

To determine the magnitude and age at PHV, we modeled the height of an individual by a population-wide growth curve plus an individual time-consistent height deviation. The model included a random warping function per individual that allowed temporal deformation of the population growth pattern, thus modeling a height development age for each subject. The models belong to the class of functional nonlinear mixed-effects models (25) and were fitted using maximum likelihood estimation. The population height curve was modeled by an increasing spline with 10 anchor points. The consistent height variations over time were modeled by a Gaussian Matérn process with smoothness 2. The time warping was done using an increasing spline with two anchor points driven by an underlying Brownian bridge. Height velocity curves were calculated from the model by differentiation of the predicted height curves. Individual ages at PHV were found as the chronological ages that corresponded to the population PHV in adjusted age. The model has been used in a previous study (22).

All statistical analyses were performed using IBM SPSS Statistics 29.0 (SPSS, USA). *P*-values <0.05 were considered statistically significant.

### Ethical considerations

All participants and their parents gave their informed assent/consent before enrollment in the study. The study was conducted according to the Declaration of Helsinki.

## Results

**Figure 1:**
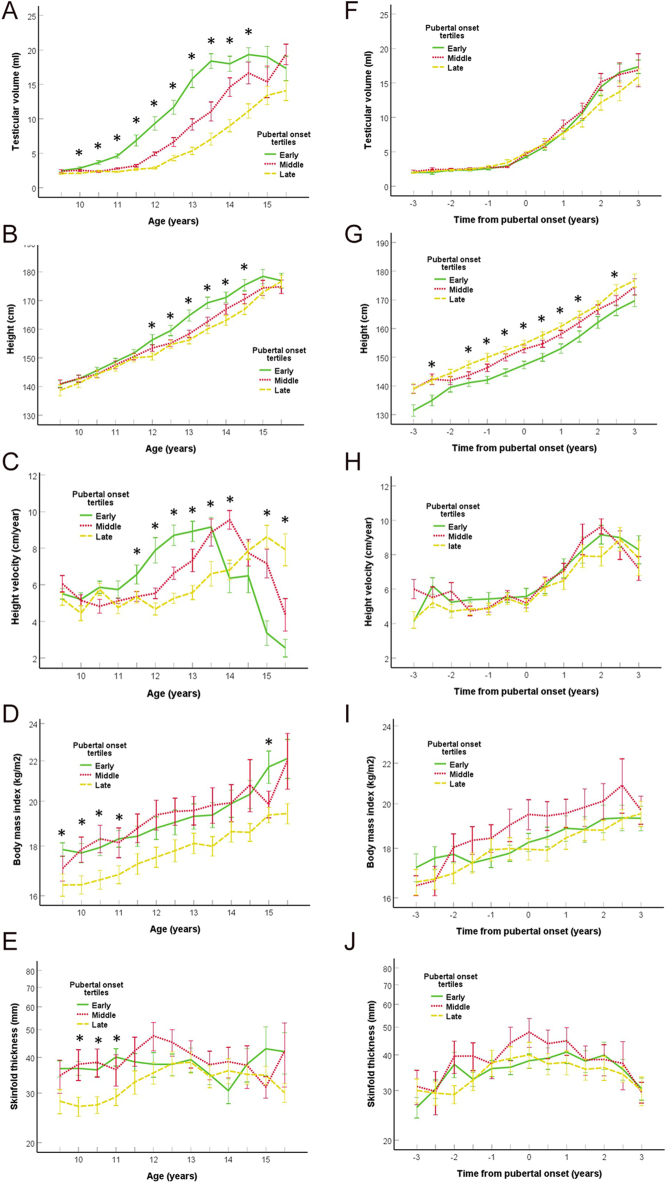
TV in milliliters (A), height in centimeters (B), height velocity in centimeters/year (C), BMI in weight/height^2^ (D) and skinfold thickness in millimeters (E) in relation to chronological age in years divided into tertile groups of early (green solid lines), middle (red dotted lines) and late (yellow broken lines) pubertal onset. TV in millimeters (F), height in centimeters (G), height velocity in centimeters/year (H), BMI in weight/height^2^ (I) and skinfold thickness in centimeters (J) in relation to pubertal onset in years (−3–3) divided into groups of early (green solid lines), middle (red dotted lines) and late (yellow broken lines) pubertal onset. Significant differences between tertile groups (*P* < 0.05) for each time point are marked with an asterisk (one-way ANOVA). The whiskers represent one standard error (SE).

The baseline characteristics of the participants are presented in Table 1. Age at pubertal onset (defined as age at first biannual examination with TV > 3 mL) was positively correlated with age at PHV (*ρ* = 0.74; *P* < 0.001) and negatively correlated with PHV (*ρ* = −0.48; *P* < 0.001). Age at PHV was negatively correlated with PHV (*ρ* = −0.55; *P* < 0.001). The duration from age at pubertal onset to age at PHV was not correlated with PHV (*ρ* = −0.12; *P* = 0.36). Longitudinal changes in TV for chronological age and for age at the onset of puberty are shown in Fig. 1A and F.

**Table 1:** Basic characteristics of all the participants divided into tertile groups of early, middle and late pubertal onset. Results are presented as means (±1.96 standard error) with the exception of overweight, which is in percentage (%). Significant differences between the three groups were evaluated by one-way ANOVA. *P* < 0.05 is expressed with an asterisk.

	Early	Middle	Late
	*n* = 21	*n* = 21	*n* = 20
Age at first examination with TV > 3 mL (years)	10.8 (±0.2)	11.9 (±0.1)	12.7 (±0.2)*
Age at onset of PH (years)	11.7 (±0.5)	11.9 (±0.4)	12.9 (±0.3)*
Age at PHV (years)	12.9 (±0.3)	13.6 (±0.3)	14.5 (±0.3)*
Pubertal duration (age at TV > 3 mL to PHV)	2.1 (±0.3)	1.7 (±0.3)	1.8 (±0.3)
PHV (cm/year)	11.0 (±0.4)	10.4 (±0.5)	10.0 (±0.3)*
At pubertal onset (age at TV > 3 mL)			
Height (cm)	147.3 (±2.4)	152.8 (±2.6)	154.7 (±2.4)*
Growth velocity (cm/year)	5.6 (±0.9)	5.2 (±0.5)	5.1 (±0.7)
Weight (kg)	39.7 (±2.3)	45.6 (±2.6)	43.1 (±2.8)*
BMI (weight/height^2^)	18.3 (±0.7)	19.5 (±1.3)	18.0 (±0.8)
SFT (mm)	38.1 (±4.1)	48.0 (±10.9)	40.0 (±8.3)
Overw./obesity – BMI z score ≥ 1 SD (%)	19	33	10

### Height and growth velocity in relation to pubertal timing

At the age of 12–14.5 years, the tertile of early-matured boys was taller than the tertile of late-matured boys (Fig. 1B), corresponding to the earlier onset of the pubertal growth spurt (Fig. 1C). Adjusted for age at pubertal onset, the tertile of early-maturing boys was shorter than the late-maturated boys throughout most of the study period (Fig. 1G). The tertile of late-maturing boys was taller (mean ± CI: 176.7 ± 4.6 cm) than the tertile of early-maturing boys (169.7 ± 4.2 cm; *P* = 0.035) at the end of the study period (3 years after pubertal onset). The tertile of early-maturing boys (0.48 (0.14–0.82) cm/year; *P* < 0.006) and the middle tertile (0.34 (0.03–0.66) cm/year; *P* = 0.034) had a significantly higher growth velocity compared with the tertile of late-maturing boys evaluated over the entire study period (Fig. 1H).

**Figure 2:**
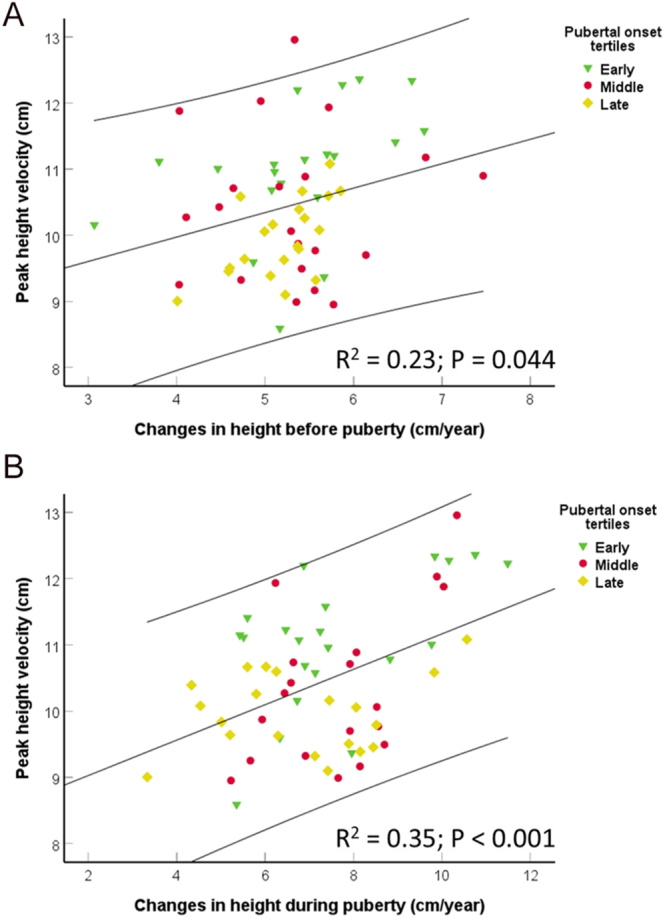
Scatterplots of PHV (cm/year) in relation to pre-pubertal (A) and pubertal (B) changes in height (cm/year) over the 2 years leading up to pubertal onset and the 2 first years after pubertal onset divided into tertile groups of early (green triages), middle (red circles) and late (yellow squares) pubertal onset. The black lines represent linear regression lines and 95% CIs between PHV and changes in height before and during puberty. Significant differences were found between PHV and tertile groups (*P* ≤ 0.006). Adjusted *R*^2^ and *P*-value by univariate analyses (ANOVA).

In cross-sectional analyses of all boys, PHV was positively associated with growth velocity at all time points from 0 to 2 years (all *P* ≤ 0.003) adjusted for age at pubertal onset (all *P* ≤ 0.052). PHV was positively associated with growth velocity in the 2 years leading up to puberty and the first 2 years of puberty, respectively (Fig. 2A and B). Growth velocity before puberty was not associated with growth velocity during puberty (*P* = 0.7).

### SFT and BMI in relation to pubertal timing and PHV

The tertile of late-maturing boys had a lower BMI and SFT than both the early tertile (mean difference ± CI, BMI: 10.8% (7.8–13.9), *P* < 0.001; SFT: 20.6% (2.2–42.2), *P* = 0.026) and the middle tertile (BMI: 6.6% (3.7–9.6); *P* < 0.001; SFT: 36.3% (6.6–48.7), *P* = 0.007) according to chronological age (Fig. 1D and E). In all boys, age at pubertal onset was negatively correlated with BMI and SFT at 9.5 and 10 years (all *ρ* ≤ −0.33, *P* ≤ 0.035). Age at PHV was negatively correlated with SFT, but not BMI, at 9.5 and 10 years (both *ρ* ≤ −0.28, *P* ≤ 0.036). However, there were no differences in BMI and SFT between early, middle and late tertiles of maturing boys adjusted for pubertal onset (both *P* ≥ 0.18) (Fig. 1I and J).

**Figure 3:**
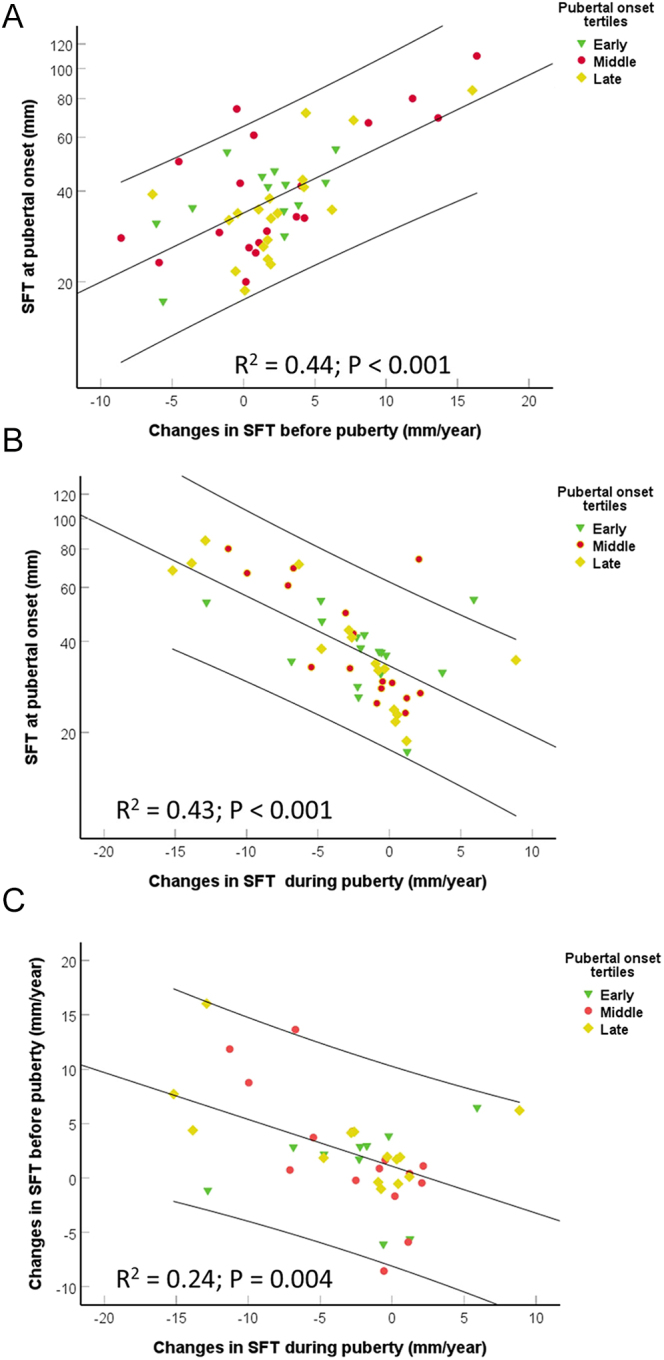
Scatterplots of sum of skinfolds (mm) at pubertal onset in relation to pre-pubertal (−3–0 years before pubertal onset; A) and pubertal (0–3 years after pubertal onset; B) changes in sum of skinfolds (mm/year). Scatterplot of changes in pre-pubertal sum of skinfolds in relation to changes in pubertal sum of skinfolds (C). The black lines represent linear regression lines and CIs. *R*^2^- and *P*-value by univariate analyses (ANOVA). The differences between the tertiles for pubertal onset were non-significant (*P* = NS).

In compiled analyses of all boys, SFT increased from 3 years before pubertal onset (0.7% (−11.0–13.9); *P* = 0.91) to a plateau at pubertal onset (22.6% (10.1–36.5); *P* < 0.001) and decreased toward 3 years past pubertal onset (set as reference) (Fig. 1I). SFT at pubertal onset was associated with the change in SFT in the 2 (*ρ* = 0.47, *P* < 0.001) and 3 years before puberty (Fig. 3A) and the first 2 (*ρ* = −0.51; *P* < 0.001) and 3 years of puberty (Fig. 3B), respectively. The increase in SFT over the 3 years before pubertal onset was associated with the decrease in SFT in the 3 years after pubertal onset (Fig. 3C). In addition, the gain in height was borderline significantly associated with the increase in SFT (*P* = 0.071) the 2 years before puberty, while the opposite was seen during the first 2 years of puberty where a lower height gain was associated with an increase in SFT (*P* = 0.005).

### Fasting insulin and IGF-1 levels in relation to pubertal timing and growth velocity

**Figure 4:**
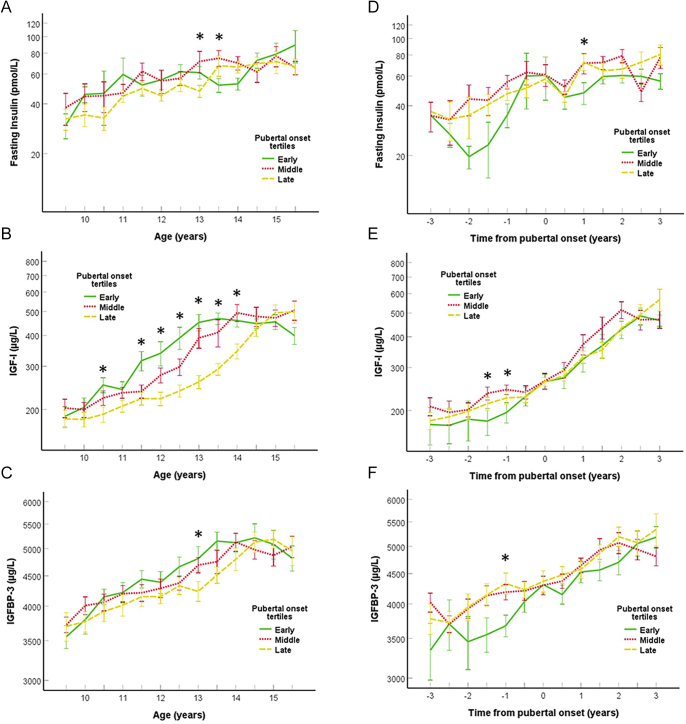
Fasting insulin levels in picomol/L (A), insulin-like growth factor 1 (IGF-1) levels in micrograms/L (B) and IGFBP-3 levels in micrograms/L (C) in relation to chronological age in years divided into tertile groups of early (green solid lines), middle (red dotted lines) and late (yellow broken lines) pubertal onset. Fasting insulin levels in picomol/L (D), IGF-1 levels in micrograms/L (E) and IGFBP-3 levels in micrograms/L (F) in relation to time to/from pubertal onset in years divided into tertile groups of early (green solid lines), middle (red dotted lines) and late (yellow broken lines) pubertal onset. Significant differences between tertile groups (*P* < 0.05) for each time point are marked with an asterisk (one-way ANOVA). The whiskers represent one standard error (SE).

Neither IGF-1 levels (*P* = 0.06) nor fasting insulin levels (*P* = 0.18) were significantly higher in the early tertile compared with the tertile of late-maturing boys according to chronological age for the entire study period (Fig. 4A and B). However, when the same analysis was done between 10 and 14 years of age, the tertile of early-maturing boys had significantly higher IGF-1 levels than the tertile of late-maturing boys (30.2% (14.0–48.6), *P* < 0.001). Neither pre-pubertal IGF-1 nor fasting insulin levels at 9.5 and 10.0 years were correlated with age at pubertal onset or age at PHV (all *P* ≥ 0.16). However, the tertile of early-matured boys had lower fasting insulin levels (−18.5% (−5.5–−29.5), *P* = 0007) than the tertile of late-matured boys (Fig. 4D) after adjustment for pubertal onset. IGF-1 levels were similar between the early, middle and late tertiles of maturing boys after adjustment for pubertal onset (Fig. 4E).

**Figure 5:**
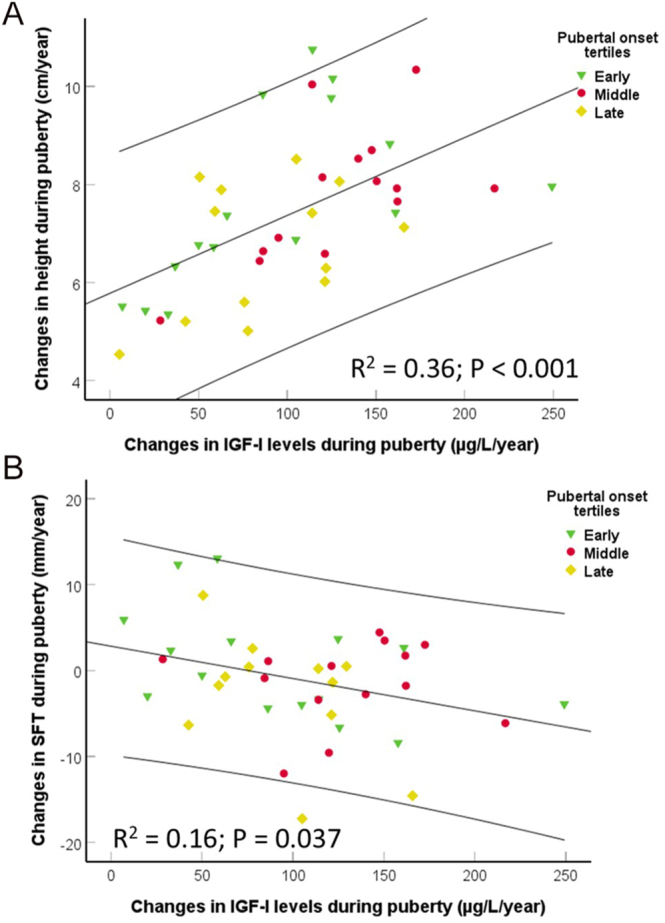
Scatterplot of changes in height (A) and skinfold thickness during puberty (B) in relation to changes in IGF-1 levels in the first 2 years of puberty. No significant difference was found between tertile groups of pubertal onset (*P* = NS). The black lines represent the linear regression lines and 95% CIs. *R*^2^- and *P*-value by univariate analysis (ANOVA).

In compiled analyses of all boys, IGF-1 levels were not associated with growth velocity before puberty. However, the gain in height was associated with the increase in IGF-1 levels during the first 2 years of puberty (Fig. 5A). In addition, the changes in IGF-1 levels were associated with the decline in SFT during the first 2 years of puberty (Fig. 5B).

Fasting insulin levels were not associated with PHV, pre-pubertal or pubertal growth velocity, neither in cross-sectional nor in longitudinal analyses (results not shown).

## Discussion

In the present longitudinal study of healthy Caucasian boys, we confirmed that early age at pubertal onset was associated with early age at PHV and a higher PHV. However, the tertile of late-maturing boys achieved a higher near-final height due to the longer pre-pubertal growth period. The IGF-1 levels did not differ between tertiles of early, middle or late timing of pubertal onset after adjusting for pubertal onset. In all boys, a higher increase in IGF-1 levels predicted an increase in growth velocity during the first two years of puberty.

Age at pubertal onset has declined over the past decades concomitantly with increased adult height. Although the secular trend toward earlier maturation (1, 2, 3, 4) and increased adult height (5, 6) tend to be more pronounced in females, the same tendency is seen in males. In accordance with previous studies (8, 9), we confirmed that earlier age at pubertal onset and age at PHV were associated with a greater magnitude of PHV in boys. The tertiles of early- and middle-maturing boys had a greater growth velocity in the entire 6-year study period compared with the tertile of late-maturing boys. In addition, the gain in height in the 2 years leading up to puberty and the first 2 years of puberty was both significantly associated with a higher PHV and earlier timing of puberty. Although the growth velocity was increased in the earlier-maturing boys, the duration from pubertal onset to PHV was similar between the early, middle and late tertiles of maturing boys. Thus, the increased growth velocity and the higher PHV did not fully compensate for the shorter total growth period in the tertile of early-maturing boys compared with the tertile of late-maturing boys. This is in contrast to findings in girls, where the shorter total growth period associated with early puberty is compensated by longer pubertal periods and increased growth velocity both before and during puberty, thereby not affecting final adult height (10, 11).

Adiposity is linked to earlier age at pubertal onset, although a U-formed relationship has been suggested (26). In the present study, pre-pubertal SFT was associated with early age at pubertal onset and early age at PHV, linking increased pre-pubertal subcutaneous fat mass to early timing of puberty as previously shown (27, 28). When adjusted for pubertal onset, SFT rose in a similar pattern toward start of puberty, after which SFT declined to baseline with no difference between maturational groups. Thus, although increased adiposity is related to early pubertal timing even in normal-weight children, they end up having a similar degree of adiposity later in puberty.

Another interesting finding is the growth-promoting properties of increasing fat mass. In the present study, the pre-pubertal increase in SFT was borderline associated with the gain in height, and the decreasing SFT during puberty was significantly associated with the gain in height. This aligns with previous findings showing that a higher BMI and SFT are linked to accelerated growth before puberty, followed by a blunted pubertal growth spurt, ultimately resulting in a non-significant impact on final height (20, 21).

Pre-pubertal IGF-1 levels at ages below 10.0 years were not associated with age at pubertal onset as previously shown (29), although a recent study contradicts these findings (16). When adjusted for pubertal onset, the IGF-1 levels were identical between the three maturational groups of boys, indicating that IGF-1 levels, like fat mass, were left-centered, but not higher *per se*. Although controversy exists on IGF-1 as a major player in the initiation of pubertal onset in boys, IGF-1 is of paramount importance to linear growth during puberty (12, 30). In our study, the IGF-1 levels were associated with growth velocity during the steepest increase in both IGF-1 levels and growth velocity during puberty (0–2 years after pubertal onset). In addition, the gain in height was predictive of the increase in IGF-1 levels over the first 2 years of puberty. This is in accordance with a study showing that IGF-1 levels at 5 years of age were predictive of the subsequent pre-pubertal growth in height in boys and girls (15). Thus, the relative level of circulating IGF-1 and the increase in IGF-1 are both associated with gain in height in children.

In addition to the growth-promoting properties, IGF-1 also increases muscle mass and reduces body fat percentage (12). In our study, increasing IGF-1 was associated with a decrease in SFT during the 2 first years after puberty. Thus, our study supports that pubertal IGF-1 increases height and simultaneously reduces subcutaneous fat mass.

Fasting insulin levels were significantly lower in the tertile of early-maturing boys compared with the two later groups of maturation when adjusted for pubertal onset. This contradicts the findings from previous studies in girls in which higher pubertal fasting insulin levels were associated with early age at breast development (11), early age at menarche (19) and trajectories of higher fasting insulin levels from birth to early childhood with early age at menarche (31). In addition, fasting insulin levels in boys were not associated with growth velocity in the present study, which has recently been shown for pubertal girls (11). This indicates that boys do not become increasingly insulin resistant at puberty as a result of early pubertal timing, to counterbalance the loss in pre-pubertal height associated with being early in puberty.

### Strengths and limitations

The strengths of the current study are the longitudinal design with biannual evaluation of pubertal stages, including TV by orchidometer and pubic hair by direct inspection; growth parameters; estimation of subcutaneous fat mass by SFT; and fasting blood sample over the course of 6 years spanning pubertal onset. This enables evaluation of changes in growth velocity, adiposity, fasting IGF-1 and insulin levels corrected for pubertal onset. In addition, all clinical examinations were done by the same three physicians throughout the study. Limitations include that fasting glucose was only determined on the first four rounds of the study, which made it impossible to calculate surrogates of insulin sensitivity throughout the study period. However, in a cohort of mostly normal-weight euglycemic children, the fasting insulin level is the best proxy of insulin sensitivity. In addition, our cohort consists of Caucasian Danish boys from socioeconomically affluent families, which could have an influence on pubertal timing and adiposity. Despite this, we believe that the study is generalizable to broader populations.

## Conclusion

In summary, the tertile of early-maturing boys had a higher growth velocity and PHV compared with the tertile of late-maturing boys, but not enough to fully compensate the loss in height due to the shorter total growth period. Pubertal timing was not associated with IGF-1 levels. IGF-1 levels were associated with a higher growth velocity and lower SFT during puberty. Thus, IGF-1, but not fasting insulin, levels seem to have an impact on growth velocity and body composition, but not pubertal timing, in healthy boys.

## Declaration of interest

The authors declare that there is no conflict of interest that could be perceived as prejudicing the impartiality of the work reported.

## Funding

Anders Juul received support from the EU HORIZON (Health) project: early prevention of obesity (eprObes) (grant agreement no. 101080219).

## Ethical considerations

The study was approved by the local ethical committee (KF 01 282214) and the Danish Data Protection Agency (2010-41-5042).
